# Comparing Long-Term Survival Outcomes for Muscle-Invasive Bladder Cancer Patients Who Underwent with Radical Cystectomy and Bladder-Sparing Trimodality Therapy: A Multicentre Cohort Analysis

**DOI:** 10.1155/2022/7306198

**Published:** 2022-05-14

**Authors:** Junlan Qiu, Haifeng Zhang, Dongkui Xu, Lin Li, Lingkai Xu, Yiqing Jiang, Tao Wen, Shun Lu, Fang Meng, Lin Feng, Xiaochen Shu

**Affiliations:** ^1^Department of Oncology and Hematology, The Affiliated Suzhou Science and Technology Town Hospital of Nanjing Medical University, Suzhou 215153, China; ^2^Department of Epidemiology, School of Public Health, Soochow University, Suzhou 215123, China; ^3^VIP Department, National Cancer Center/National Clinical Research Center for Cancer/Cancer Hospital, Chinese Academy of Medical Sciences and Peking Union Medical College, Beijing 100021, China; ^4^Department for Communicable Disease Control and Prevention, Suzhou Wuzhong Center for Disease Prevention and Control, Suzhou 215128, China; ^5^Department of General Surgery, Harrison International Peace Hospital, Hengshui 053000, China; ^6^Medical Research Centre, Beijing Chao-Yang Hospital, Capital Medical University, Beijing 100020, China; ^7^Department of Radiation Oncology, Sichuan Cancer Hospital/Institute, University of Electronic Science and Technology of China, 610041 Chengdu, China; ^8^Centre of Systems Medicine, Chinese Academy of Medical Sciences, Beijing 100730, China; ^9^Suzhou Institute of Systems Medicine, Suzhou 215123, China; ^10^Jiangsu Key Laboratory of Preventive and Translational Medicine for Geriatric Diseases, Medical College of Soochow University, Suzhou 215123, China

## Abstract

**Background:**

Although radical cystectomy (RC) is the clinical practice guideline-recommended treatment of muscle-invasive bladder cancer (MIBC), bladder-sparing trimodality therapy (TMT) has emerged as a valid treatment option. Findings comparing the survival outcomes for MIBC patients who underwent RC and TMT are inconclusive.

**Objective:**

We designed a large hospital-based multicohort study to compare the effectiveness of TMT with RC.

**Methods:**

Information on deaths was jointly retrieved from EMR (electronic medical record), cause of death registry, and chronic disease surveillance as well as study-specific questionnaire. To avoid the systematical difference between patients who received two modalities, RC-MIBC cohort was propensity score-matched to TMT-MIBC cohort, and the Cox proportional hazard regression was used to calculate the overall survival (OS) and disease-specific survival (DSS).

**Results:**

There were 891 MIBC patients treated with RC and another 891 MIBC patients who underwent with TMT in the propensity score matching. Comparable effectiveness between two modalities was observed for DSS (HR, 1.20; 95% confidence interval (CI), 0.94 to 1.49) and OS (HR, 1.17; 95% CI, 0.91 to 1.43) according to multiple adjustment after a median follow-up of approximately 9.3 years. However, a relatively higher mortality rate around 5 years after TMT treatment was found compared to RC (HR, 1.26; 95% CI, 1.01 to 1.53). The respective 5-year OS rates were 69% and 73% for TMT cohort and RC cohort, respectively.

**Conclusions:**

Our findings supported that MIBC patients with TMT yielded survival outcomes comparable to MIBC patients who underwent RC overall. Treatment options should be suggested considering patients' age and willingness.

## 1. Introduction

The International Agency for Research on Cancer (IARC) updated an estimate of 573,278 bladder cancer new cases and 212,536 bladder cancer deaths in 2020 worldwide in the latest report on the global cancer burden using the GLOBOCAN 2020 [1]. In US, the estimated bladder cancer new cases and bladder cancer deaths were 83,730 and 17,200 in 2021, respectively. A male predominance of new bladder cancer cases and deaths was consistently observed across different countries and regions [2].

Chemotherapy followed by radical cystectomy (RC) with extended pelvic lymphadenectomy is the guideline-recommended treatment of muscle-invasive bladder cancer in China [3]. Although it is a lethal disease, only around one-fifth of MIBC patients received surgery [4, 5]. Considering the nonnegligible morbidity and mortality associated with RC, clinicians and patients have sought alternative treatments for patients being older and having increased comorbidities [6]. In recent years, the use of bladder-preserving trimodal therapy has increased [5]. The current NCCN (National Comprehensive Cancer Network) Clinical Practice Guidelines indicate that bladder-preserving therapy is a safe and effective alternative to RC for MIBC. The less-invasive trimodal “bladder-sparing” approaches that combine maximal transurethral resection, chemotherapy, and radiotherapy to treat muscle-invasive bladder cancer has been used in clinics in recent years [3]. Several organizations, including the American Urological Association and the European Association of Urology, have updated their guidelines to support the use of radiotherapy combined with chemotherapy in selected patients with muscle-invasive disease.

To date, data comparing the survival outcomes for MIBC patients underwent RC and TMT are still limited and the findings are inconsistent. The absence of evidence from randomized clinical trials makes the results from observational studies valuable [7]. A hospital-based follow-up study compared the survival of chemoradiation and salvage radical cystectomy for MIBC after TMT and concluded that TMT was an alternative to radical cystectomy for patients with MIBC [8]. In another study, the effectiveness of radical cystectomy and bladder-sparing trimodality therapy on patients with MIBC was compared from a total of 112 patients after propensity score matching, and the 5-year DSS (disease-specific survival) rate was similar in the RC (73.2%) and TMT (76.6%) groups (*P* = 0.49) [9]. A Swedish nationwide population-based cohort study [10] reported a higher risk of death for patients treated with radiotherapy in comparison with those underwent with RC (HR: 1.5-1.6) based on 3309 patients. The imbalance of baseline characteristics that is radiotherapy group were older and had more advanced comorbidity may weaken this finding. A population-based cohort study used data from the Surveillance, Epidemiology, and End Results-Medicare-linked database and observed that patients who underwent trimodal therapy had significantly decreased overall survival (hazard ratio (HR), 1.49; 95% CI, 1.31-1.69) and cancer-specific survival (HR, 1.55; 95% CI, 1.32-1.83) after a 1 : 1 propensity score matching [5]. The National Cancer Database comparison of RC vs. TMT found that overall survival was no longer significantly different between RC (3 years 52.1% and 5 years 41.0%) and TMT (3 years 53.3% and 5 years 40.1%) after matched pair analyses [11]. More recently, an update based on more cases from the National Cancer Database reported inconsistent results that overall survival was significantly shorter in the CMT (chemoradiation) group than in the RC group in both multivariate analysis (HR 1.15, 95% CI 1.08-1.22; *P* < 0.001) and propensity score-weighted analysis (HR 1.18, 95% CI 1.07-1.30; *P* < 0.001) [12].

Given this gap of the literature, we designed a hospital-based multicentre cohort to compare the overall- and cause-specific death of radical cystectomy with those of trimodal therapy. Also, we used a propensity score-adjusted direct comparison of TMT to RC in MIBC to better balance the imbalance of covariates at baseline.

## 2. Materials and Methods

### 2.1. Study Population

The present retrospective cohort study was a part of an ongoing cohort study which was named as CaPRICE (Cancer Prognosis and Recurrence multICohort Examination: Make CaPRICE predictable!) study. Study participants in this study were recruited in four independent tertiary hospitals in China: Beijing Chao-Yang Hospital, Sichuan Cancer Institute/Hospital, the Affiliated Suzhou Science and Technology Town Hospital of Nanjing Medical University, and Harrison International Peace Hospital. More details were also depicted in our previous publications [13, 14]. Our study was performed with the approval of the institutional review board (Q413900215). The need for informed consent was waived in view of the retrospective observational nature of the study and the anonymity of the data. We retrospectively reviewed the electronic medical records (EMR) of patients whose first diagnosis of cancer was urinary bladder malignancy according to International Classification of Disease for Oncology, 3rd Edition (ICD- O-3) site codes C67.0 to C67.9 from January 31, 2005, to December 31, 2014. Clinical staging for all cases was assessed. Muscle-invasive bladder cancer (MIBC) was defined as localized bladder cancer clinically staged as cT2–T4M0. Only relatively older MIBC patients aged between 60 and 79 were finally included. Patients codiagnosed with any other cancers (ICD10: C00-C97 exclusive of C67.0 to C67.9), multiple/unknown primary, and missing clinical stage and treatment information were excluded.

All patients received treatment, either radical cystectomy (RC) or trimodal therapy (TMT), during 2005-2014. RC was defined as radical cystectomy; radical cystectomy plus either ileal conduit, continent reservoir/pouch, abdominal pouch, or in situ pouch; or radical cystectomy including anterior exenteration, posterior exenteration, or total exenteration (surgery to primary site code 60-74). The radical cystectomy group comprised patients who underwent only surgery or surgery in combination with radiotherapy or chemotherapy. The TMT group consisted of patients who underwent transurethral resection of the bladder followed by radiotherapy and chemotherapy. TMT was identified by diagnosis for both radiotherapy and chemotherapy in the absence of a concomitant treatment for radical cystectomy. Also, patients with RC after the TMT were excluded.

### 2.2. Ascertainment of Study Cohorts and Propensity Score Matching at Baseline

Any MIBC patient who underwent radical cystectomy were identified and followed up in RC-MIBC cohort (radical cystectomy muscle-invasive bladder cancer cohort). TMT-MIBC cohort (trimodal therapy muscle-invasive bladder cancer cohort) was composed of MIBC patients who underwent treatment of bladder-sparing trimodal therapy.

**Figure 1:**
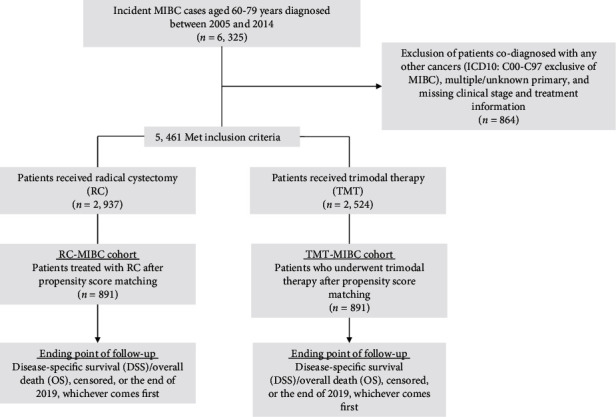
Flow diagram of study participants.

Patients who received radical cystectomy may systematically differ from patients who received trimodal therapy. For example, patients who are older, have greater comorbidities, and have a lower stage of cancer may be more likely to receive trimodal therapy. We used propensity score matching to control selection bias and confounding while comparing 2 treatments. Multiple logistic regression analyses were used to control for the imbalance due to the confounding difference between RC-MIBC cohort and TMT-MIBC cohort by performing propensity score matching. Based on a multiple conditional logistic regression model including covariates age, gender, year at treatment, education achievement, clinical T stage, and Charlson Comorbidity Index (CCI), the propensity score that was the probability of RC of an individual conditional on a serial of observed demographic and clinical characteristics was calculated and we selected to use nearest neighbour propensity score matching. TMT-MIBC cohort was matched to RC-MIBC cohort with the minimum difference in propensity score. The final RC-MIBC cohort and TMT-MIBC cohort consisted of 891 and 891 patients, respectively (Figure 1).

### 2.3. Data Collection and Interview

#### 2.3.1. Sources of Data and Covariates

Causes of death have been accessed by using the following ways in combination: cause of death reporting system from Centre for Disease Control and Prevention (CDC) in each region, EMR system in hospitals, death certificate, maternal and child surveillance system, and cancer registry data. Information of patients that was still unavailable by combining ways above was collected by phone interview, and these patients comprised less than 5% of all.

In addition, demographic and clinical characteristics were collected by a combined retrieval of medical records and other above-mentioned data sources, supplemented by a survey using a structured questionnaire. The questionnaires were administered by trained interviewers. A predesigned and validated questionnaire was used to collect information on related factors. Tumor characteristics included histology, T stage, and N stage, with TNM stage according to the AJCC staging system. The Charlson Comorbidity Index (CCI) assesses comorbidity level by taking into account both the number and severity of 19 predefined comorbid conditions, which provided a weighted score of a client's comorbidities. In this study, CCI was recorded as 0, 1, and 2 or more.

#### 2.3.2. Follow-Up and Study Outcomes

Starting date of the study was the date of treatment (RC or TMT), and ending date of the study was date of death, emigration, or 31 December 2019, whichever came first. Time in years from diagnosis was used as timescale in all analyses. We identified all MIBC patients who died during January 1, 2005, to December 31, 2019, to make sure that each participant in the entire cohorts had at least five years of follow-up. The primary outcome was overall survival (OS) from the initial diagnosis to the date of death or last follow-up. Overall survival (OS) included death from all the causes (ICD-10 code A00-Z99), while cause-specific survival (CSS) referred to cause of death due to bladder cancer (ICD-10 code C67.0 to C67.9).

### 2.4. Statistical Analyses

Observational studies that mimic results from a randomized controlled trial must control for selection bias to properly estimate the effect of treatment. We used propensity score matching as a pseudorandomization and made 2 treatment groups comparable. The Kaplan-Meier (K-M) model was used to calculate the probability of disease-specific survival (DSS) and the probability of overall survival (OS) as a function of time. The differences between the K-M curves were tested for significance by the log-rank test. Cox PH regression was used to estimate hazard ratios (HR) and its 95% confidence interval (CI) for bladder cancer mortality and overall mortality. All baseline covariates were well balanced in the propensity score-matched sample. Therefore, only treatment information was controlled for in the final Cox PH regression model.

Using the R packages (version 4.1.0, R: a language and environment for statistical computing, R Foundation for Statistical Computing, Vienna, Austria; URL http://www.R-project.org/.), the K-M survival curves were drawn and long-rank test was analyzed to test the overall OS/DSS difference. Furthermore, we used the “ComparisonSurv” (Lyu, 2020) in R packages to further check the difference between two treatments for fix point by fix point test. All other analyses were performed using the SAS statistical package ver. 9.4 (SAS Institute Inc., Cary, NC). All *P* values were based on two-sided tests, with the statistical significance level set to 0.05.

### 2.5. Ethical Statement

Our research protocols were approved by the institutional review boards of Soochow University and the respective ethical committees at the participating hospitals (Q413900215). The implementation of the current study adhered to the tenets of the Declaration of Helsinki of the World Medical Association with regard to scientific research on human subjects. In order to preserve anonymity, individual record of all participants was deidentified before the analysis.

## 3. Results

This multicohort study was totally comprised of 6,325 incident muscle-invasive bladder cancer (MIBC) patients aged 60-79 years, who were diagnosed between 2005 and 2014 at four tertiary hospitals in different cities in China. Among them, 5,461 patients met the inclusion criteria and were included in the further analysis. In order to eliminate the systematical difference between two modalities (radical cystectomy (RC) vs. bladder-sparing trimodality therapy (TMT)) for MIBC patients, 891 MIBC patients who underwent RC were identified and matched to the MIBC patients treated with TMT by propensity score at a ratio of 1 : 1 to control the selection bias and confounding between two cohorts at baseline (Figure 1). After a mean of 9.4 years and 8219 accumulated person-years of follow-up, we totally observed 635 deaths in the TMT cohort, among which 351 died of MIBC diagnosis. Comparatively, there were a mean of 9.2 years and 8304 person-years accumulated during the follow-up for the reference cohort (RC-MIBC patients), with a total of 546 overall death and 295 MIBC death. We did not observe statistical difference as for overall survival (OS) rates (*P* > 0.05) between patients who underwent two modalities, neither were the disease-specific survival (DSS) rates between treatment cohorts (*P* > 0.05).

Table 1 shows the main demographic and clinicopathological characteristics as well as treatment information for MIBC patients who underwent two different modalities. All baseline covariates were well balanced in the propensity score-matched sample, and the systematical difference from patients between RC and TMT groups was eliminated (*P* > 0.05). In our study sample, MIBC patients were more likely to be observed among men (over 70%), those with no university education (over 85%), and at the middle staging (T2-T3a). Charlson Comorbidity Index (CCI) above 2 accounted for less than 20% in both modality groups. The highest proportion of highest education achievement was elementary school, followed by secondary school and university and above. Two-thirds of the MIBC patients were experiencing a clinical stage of T2 at the time of MIBC diagnosis. More MIBC patients accepted neoadjuvant chemotherapy and less accepted adjuvant chemotherapy before RC than before TMT (*P* < 0.001). No significant difference was observed for response to chemoradiation by modality (*P* > 0.0.5).

**Figure 2:**
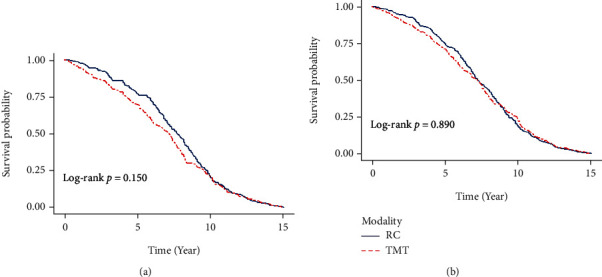
(a) Probability of disease-specific survival (DSS) of MIBC patients who underwent TMT in comparison with MIBC patients treated with RC. (b) Probability of overall survival (OS) of MIBC patients who underwent TMT in comparison with MIBC patients treated with RC.

Overall, comparable effectiveness was observed between two modalities. MIBC patients treated with bladder-sparing trimodality therapy were associated with an elevated but not statistically significant HR of 1.20 (95% CI, 0.94 to 1.49) for MIBC death and 1.17 (95% CI, 0.91 to 1.43) for overall death (Table 2). Moreover, the trend of survival was observed to be impacted by length of follow-up, that is, relatively higher survival rates among RC for the short-term follow-up compared to similar survival rates for the long-term follow-up, with the crossover point between 5 and 10 years. Similar trends were noted for DSS, too. The K-M survival curves based on the modality groups were plotted for MIBC-specific survival (Figure 2(a)) and overall survival (Figure 2(b)). The association was further stratified by age at diagnosis of MIBC, gender, year at treatment, clinical T stage, and Charlson Comorbidity Index (CCI) (Table 2). The increment of mortality risk was observed to be relatively more prominent among later diagnosis of MIBC, more comorbidities, and later clinical stage.

As shown in Table 3, we evaluated how the use of neoadjuvant chemotherapy (NAC) or adjuvant chemotherapy (AC), radiation dose, and response to chemoradiation impacted the survival rates of MIBC patients. We observed a series of slight mortality risk variation. A relatively lower mortality rates were observed for those who received neoadjuvant chemotherapy (NAC) or adjuvant chemotherapy (AC) for both RC and TMT treatment compared with those who did not have NAC or AC. Comparatively high dose of radiation and complete response to chemotherapy seems to have reduced risk of mortality although significant associations were not observed.

In Table 4, impact of length of follow-up on survival rates was accessed. Compared to RC-MIBC cohort, survival rates in TMT cohort were observed to be varied by length of follow-up: relatively lower survival rates among those underwent TMT during relatively shorter follow-up and similar and even higher survival rates for patients after TMT in long-term follow-up periods.

## 4. Discussion

In this multicentre retrospective cohort study, we totally identified 5,461 MIBC patients who met the inclusion criteria. Based on a propensity score sampling, a comparable effectiveness of both modalities, i.e., RC vs. TMT, on MIBC survival rates was observed. Our study further demonstrated a relatively higher survival rates among RC-MIBC cohort with reference to TMT-MIBC cohort after propensity score matching during the first half follow-up and a similar survival rates during the second half follow-up for both OS and DSS. Moreover, elevation of mortality risk was observed to be relatively more prominent among earlier diagnosis of MIBC, more comorbidities, and later clinical stage. Notably, the crossover point for the survival of the MIBC patients who underwent RC and TMT was after the middle of follow-up.

To date, data directly comparing the survival outcomes for MIBC patients who underwent RC and TMT are still limited and the findings are inconsistent. No randomized clinical trials have compared RC and bladder-sparing modality with TMT; the recent United Kingdom phase III trial, selective bladder preservation against radical excision (SPARE), unfortunately failed to accrue patients and resulted in premature closure [15]. Large cohort study generated conflicting results. Comparative effectiveness study based on SEER (The Surveillance, Epidemiology, and End Results-Medicare database) registry reported inconsistent results between these two modalities. One study reported that BPT (bladder-preserving therapy) was associated with an increased hazard of death from any cause (hazard ratio (HR) 1.26; 95% confidence interval (CI) 1.05-1.53) and from bladder cancer (HR 1.31; 95% CI 0.97-1.77) [16]. More recently, another population-based cohort study used SEER data and observed that patients who underwent trimodal therapy had significantly decreased overall survival (hazard ratio (HR), 1.49; 95% CI, 1.31-1.69) and cancer-specific survival (HR, 1.55; 95% CI, 1.32-1.83) after a 1 : 1 propensity score matching [5]. By contrast, another retrospective, observational cohort study using data from the Surveillance, Epidemiology, and End Results (SEER)-Medicare database in which propensity score-based multivariable adjustment, instrumental variable analysis, and simulations were used reported a similar survival outcomes (hazard ratio (HR), 0.94; 95% CI, 0.55-1.18) between RC and TMT. This study included 1843 patients, of whom 1426 treated with RC and 417 underwent CMT [16]. A Swedish nationwide population-based cohort study reported a higher risk of death for patients treated with radiotherapy in comparison with those underwent with RC (HR: 1.5-1.6) based on 3309 patients [10]. The imbalance of baseline characteristics that is radiotherapy group were older and had more advanced comorbidity may weaken this finding, while US National Cancer Database comparison of RC vs. TMT found that overall survival was no longer significantly different between RC (3 years 52.1% and 5 years 41.0%) and TMT (3 years 53.3% and 5s year 40.1%) after matched pair analyses [11]. However, an update based on more cases from the National Cancer Database reported inconsistent results that overall survival was significantly shorter in the CMT (chemoradiation) group than in the RC group in both multivariate analysis (HR 1.15, 95% CI 1.08-1.22; *P* < 0.001) and propensity score-weighted analysis (HR 1.18, 95% CI 1.07-1.30; *P* < 0.001) [12]. NCB does not report disease-specific survival, so OS was the only outcome that could be included in the analysis.

Single-center studies have mostly reported comparable survival outcomes [9, 17]. A single-center study conducted in Massachusetts General Hospital found a favorable long-term OS and DSS with bladder preservation patients in a cohort of 475 patients after a median follow-up of 4.55 years and concluded that TMT could be an alternative to RC for MIBC patients [8]. Data from MIBC patients treated in a multidisciplinary bladder cancer clinic (MDBCC) from 2008 to 2013 showed that TMT yielded midterm survival outcomes similar to those of MIBC patients who underwent RC after propensity score matching based on a total of 112 patients. The 5-year DSS rate was 73.2% and 76.6% in the RC and TMT groups, respectively (*P* = 0.49) [9]. Authors stated that multidisciplinary consultation with experts could lead to improvement in BC staging, which may optimize treatment recommendations. Stage was used as a surrogate for appropriate treatment delivery. It was reported that incorrect clinical staging, particularly understaging, had been identified as a serious problem in bladder cancer and MDBCC assessment resulted in treatment changes for 33% of referred patients [9, 18]. Similar 5-year DSS for MIBC patients who underwent RC or TMT was also reported in other studies [19, 20]. Pooled analysis of most bladder preservation studies (prospective RTOG bladder-preserving CMT protocols) demonstrated long-term DSS comparable to modern immediate cystectomy studies, for patients with similarly staged MIBC. Six historical radiation therapy oncology group single-arm studies demonstrated a similar 5-year DSS rate of 71% compared with modern MIBC RC modality. Given the low incidence of late recurrences with long-term follow-up, CMT can be considered as an alternative to radical cystectomy, especially in elderly patients not well suited for surgery [21].

In consideration of the substantial differences at baseline characteristics between patients who chose either RC or TMT in most of present observational reports, many of these studies used propensity score (PS) sampling or instrumental variable (IV) method to avoid this imbalance and treatment selection bias as well. The results from several NCDB (US National Cancer Database) matched paired analyses showed a comparable survival rate between cystectomy/chemo vs. chemoRT consistently [9, 11, 22], while a recently published NCDB analysis reported that overall survival was better for RC than definitive chemoRT as radiation doses ≥ 40 Gy and the underlying reason was elucidated [23]. Author used SEER data to compare survival after treatment and observed that MIBC patients who underwent RC had a better survival using unmatched comparison, and survival difference was not found after considering upstaging in a multivariate Cox PH regression [16]. Radiation dose could be another risk factor which confounded the association observed. The results using the NCDB showed that a radiation dose response as ≥60 Gy resulted in better overall survival than 55 Gy to <60 Gy [11]. Like propensity score method, IV method could adjust for both observed and unobserved confounding effects. A large Canadian cohort study of 5259 patients observed no disparity in DSS for MIBC patients treated with radiotherapy when compared to RC in a propensity score sample, with a slightly elevated OS after a follow-up of 5 years for patients who underwent with radiotherapy [24]. In other study where instrumental variable (IVs) analyses were used, both similar survival [10, 16] and favored survival for patients underwent with RC were reported [4].

Impact of treatment information was investigated in the present study. In our study, 17.2% patients who accepted RC had been received NAC (neoadjuvant chemotherapy) before the treatment. We observed that those accepted NAC before treatment in TMT cohort were found to have lower hazard ratios (HR, 1.10; 95%, 0.51-2.03) compared with those who did not underwent NAC before treatment in TMT cohort (HR, 1.18; 95%, 0.90-1.48) when compared to RC cohort, which was in line with previous report and guideline recommendations as well [5, 25]. Previous study reported that AC (adjuvant chemotherapy) was associated with improved survival in patients with locally advanced bladder cancer (HR, 0.70; 95%, 0.64-0.76) [4]. The insignificant results in the present study could be due to the reduced sample size from stratified analyses or as a result of limited cases accepted NAC before treatment. Data from SEER-Medicare database on MIBC treatment showed that a disturbing 51% of patients did not receive any definitive therapy [4]. Response to chemoradiation and clinical T stage could be significant predictors for both OS and DSS, while in our study, these two factors were balanced between two modality groups.

In this study, we also investigated the effect of follow-up period on OS and DSS for MIBC patients who underwent two modalities. Overall, we observed a comparable survival rates between RC and TMT for the whole follow-up period, together with the similar survival in the 1-year and 10-year follow-ups. However, a relatively higher mortality rate around 5 years after TMT treatment was found compared to RC (HR, 1.26; 95% CI, 1.01 to 1.53). The effect of modalities, RC or TMT, on the survival of MIBC was inconsistent in literatures. A recent study using the data from US National Cancer Database observed that the mortality rate was relatively higher after RC than after CMT during the first 12 months although their results favored RC, which was elucidated as that could be partially related to the higher complication and mortality rates after RC [12]. In our study, we used a propensity score sampling to compare the survival between two modalities in which the complication and CCI were balanced. In a setting of a multidisciplinary bladder cancer clinic, authors observed that TMT provided midterm survival outcomes comparable to RC in a propensity score sample [9]. A meta-analysis based on 8 clinical studies totally observed a statistically significant difference at more than 10-year OS between RC and TMT, with relatively higher OS and DSS and lower mortality rate for MIBC patients who underwent RC compared to TMT [26]. Authors also observed that based on 5 studies with follow-up time more than 10 years, RC was superior to TMT regardless of OS or DSS rates [26]. By contrast, another meta-analysis reported that TMT generated outstanding 5-year OS rates among these two interventions [27]. However, a long-term comparable effectiveness as for OS and DSS rates between RC and TMT was also reported in another systematic review [28]. The 10-year OS rates of 45.6% and 36-39% were, respectively, observed for patients who were treated with RC [29] and TMT [8]. A small single-center study reported adverse association of TMT with age in older adults, which suggest that TMT may be more suitable for the elder [30]. Factors impeded the comparability of treatment effectiveness in the literatures included sample size of the study, use of propensity score matching or instrumental variable, difference by the complexity of a propensity score model and the range of variables involved, proportion of AC/NAC (neoadjuvant chemotherapy) administration, proportion of salvage cystectomy, completeness of TMT, confounds by indication, and dosage and duration of radiochemotherapy.

Limitations of this study could include its retrospective nature and the associated bias. We used propensity score matching to create comparable RC and TMT cohorts. Additionally, it may be improper to generalize our findings to younger patients. The present study has a number of strengths. First, it has the ability to combine data from multiclinics to increase sample size to acquire a better statistical power. Second, treatment information was reviewed in an unbiased fashion. Overall, short-term, middle-term, and long-term survival rates were compared, which presents joint evidence to support the clinical decision. Third, impact of comorbidities on patient survival was eliminated by including CCI (comorbidities index) into the propensity score calculation. Fourth, the follow-up for any study subjects was at least 5 years, which makes it feasible to analyze the impact of follow-up period on MIBC survival. In addition, multiple sources of outcome acquirement ensured the completeness of the data.

To sum up, this is one of the first studies examining whether the MIBC prognosis will be comparable between patients who underwent RC and those who were treated by TMT in a propensity score-matched multicentre cohort after a long follow-up, wherein our main finding is that comparable survival rates between two modalities suggest that patients eligible for TMT should be offered the opportunity to a bladder-sparing modality, especially considering the age and willingness. TMT must be beneficial to improve quality of life (QOL) and be associated with better sexual function and better body image perception compared to RC. Moreover, our study, among the first, provided additional knowledge on the relative effectiveness of RC and TMT treatment on MIBC survival according to the follow-up period: a comparable short-term/long-term survival for between modalities and a favored midterm survival for RC.

## Figures and Tables

**Table 1:** Characteristics of RC-MIBC cohort versus TMT-MIBC cohort after propensity score matching.

Characteristics	RC-MIBC cohort		TMT-MIBC cohort		*χ* ^2^	*P* value
	No.	%	No.	%		
All	891	100.0	891	100.0		
Demographic and clinicopathological characteristics						
Age at MIBC diagnosis						
60-69	494	55.4	477	53.5	0.654	0.419
70-79	397	44.6	414	46.5		
Median age (years)	68		69			
Gender						
Men	635	71.3	625	70.2	0.271	0.603
Women	256	28.7	266	29.8		
Year at diagnosis						
2005-2009	383	43.0	381	42.8	0.009	0.924
2010-2014	508	57.0	510	57.2		
Highest education achievement						
Elementary school	396	44.5	411	46.1	2.082	0.353
Secondary school	372	41.7	377	42.3		
University and above	123	13.8	103	11.6		
Clinical T stage						
T2	585	65.7	597	66.4	3.936	0.269
T3a	195	21.9	202	22.7		
T3b	58	6.5	57	6.4		
T4a	53	5.9	35	4.5		
Charlson Comorbidity Index (CCI)						
0	548	61.5	541	60.7	0.376	0.829
1	185	20.8	182	20.4		
2 or more	158	17.7	168	18.9		
Treatment information						
Neoadjuvant chemotherapy, *n* (%)						
Yes	153	17.2	36	4.0	81.022	<0.001
No	738	82.8	855	96.0		
Adjuvant chemotherapy, *n* (%)						
Yes	296	33.2	515	57.9	108.532	<0.001
No	595	66.8	376	42.2		
Radiation dose, *n* (%)						
<60 Gy	782	87.8	467	52.4	265.607	<0.001
≥60 Gy	109	12.2	424	47.6		
Response to chemoradiation, *n* (%)						
Complete	613	68.8	642	72.1	2.267	0.132
Incomplete	278	31.2	249	27.9		

The propensity score was constructed for each participant according to the following covariates: age, gender, year at diagnosis, education achievement, clinical T stage, and Charlson Comorbidity Index (CCI). RC: radical cystectomy; TMT: bladder-sparing trimodality therapy; MIBC: muscle-invasive bladder cancer.

**Table 2:** Proportional hazard regression model for OS and DSS of MIBC patients by demographic and clinical characteristics: RC vs. TMT.

Characteristics	No. of patients	Overall survival (OS)		Disease-specific survival (DSS)	
		HR	95% CI	HR	95% CI
RC-MIBC cohort	891	1.00 [reference]		1.00 [reference]	
TMT-MIBC cohort	891	1.17	0.91-1.43	1.20	0.94-1.49
Age at diagnosis					
60-69	477	1.24	0.87-1.65	1.25	0.89-1.70
70-79	414	1.08	0.71-1.53	1.10	0.73-1.57
Gender					
Men	625	1.19	0.89-1.57	1.21	0.87-1.64
Women	266	1.16	0.69-1.68	1.20	0.70-1.74
Year at treatment					
<2010	381	1.05	0.64-1.48	1.04	0.63-1.47
≥2010	510	1.27	0.96-1.51	1.28	0.97-1.61
Clinical T stage					
T2-T3a	799	1.03	0.90-1.45	1.06	0.87-1.49
T3b-T4a	92	1.36	0.98-1.87	1.39	0.97-1.99
Charlson Comorbidity Index (CCI)					
0-1	548	1.06	0.86-1.50	1.08	0.85-1.53
2 or more	343	1.21	0.83-1.62	1.23	0.81-1.66

Analyses were based on the Cox PH regression. All baseline covariates were well balanced in the propensity score-matched sample according to demographic and clinicopathological characteristics. Therefore, only treatment information was controlled for in the final Cox PH regression model. OS: overall survival; DSS: disease-specific survival; HR: hazard ratio; CI: confidence interval; RC: radical cystectomy; TMT: bladder-sparing trimodality therapy; MIBC: muscle-invasive bladder cancer.

**Table 3:** Proportional hazard regression model for OS and DSS of MIBC patients by treatment information: RC vs. TMT.

Characteristics	No. of patients	Overall survival (OS)		Disease-specific survival (DSS)	
		HR	95% CI	HR	95% CI
RC-MIBC cohort	891	1.00 [reference]		1.00 [reference]	
TMT-MIBC cohort	891	1.17	0.91-1.43	1.20	0.97-1.49
Neoadjuvant chemotherapy (NAC)					
Yes	36	1.10	0.51-2.03	1.08	0.45-2.14
No	855	1.18	0.90-1.48	1.19	0.86-1.53
Adjuvant chemotherapy (AC)					
Yes	515	1.08	0.88-1.47	1.09	0.86-1.50
No	376	1.21	0.77-1.69	1.23	0.74-1.71
Radiation dose					
<60 Gy	467	1.24	0.83-1.61	1.25	0.81-1.64
≥60 Gy	424	1.06	0.79-1.43	1.09	0.77-1.45
Response to chemoradiation					
Complete	642	0.99	0.72-1.32	1.00	0.73-1.50
Incomplete	249	1.34	0.68-1.71	1.36	0.64-1.80

Analyses were based on the Cox PH regression. All baseline covariates were well balanced in the propensity score-matched sample. Therefore, only treatment information was controlled for in the final Cox PH regression model. OS: overall survival; DSS: disease-specific survival; HR: hazard ratio; CI: confidence interval; CI: confidence interval; RC: radical cystectomy; TMT: bladder-sparing trimodality therapy; MIBC: muscle-invasive bladder cancer.

**Table 4:** Proportional hazard regression model for OS and DSS of MIBC patients by treatment time and treatment modality.

Time by modality		No. of patients	Overall survival (OS)		Disease-specific survival (DSS)	
			HR	95% CI	HR	95% CI
1 year	RC-MIBC cohort	891	1.00 [reference]		1.00 [reference]	
	TMT-MIBC cohort	891	1.07	0.84-1.36	1.11	0.89-1.44
5 years	RC-MIBC cohort	891	1.00 [reference]		1.00 [reference]	
	TMT-MIBC cohort	891	1.26	1.01-1.53	1.30	1.04-1.59
10 years	RC-MIBC cohort	891	1.00 [reference]		1.00 [reference]	
	TMT-MIBC cohort	891	0.92	0.71-1.26	0.99	0.80-1.35

Analyses were based on the Cox PH regression. All baseline covariates were well balanced in the propensity score-matched sample according to demographic and clinicopathological characteristics. Therefore, only treatment information was controlled for in the final Cox PH regression model. OS: overall survival; DSS: disease-specific survival; HR: hazard ratio; CI: confidence interval; RC: radical cystectomy; TMT: bladder-sparing trimodality therapy; MIBC: muscle-invasive bladder cancer.

## Data Availability

The datasets used during the present study are available from the corresponding author upon reasonable request.
